# Characterization of mesenchymal stem cells and fibrochondrocytes in three-dimensional co-culture: analysis of cell shape, matrix production, and mechanical performance

**DOI:** 10.1186/s13287-016-0301-8

**Published:** 2016-03-12

**Authors:** Mary Clare McCorry, Jennifer L. Puetzer, Lawrence J. Bonassar

**Affiliations:** Meinig School of Biomedical Engineering, Cornell University, Ithaca, NY USA; Sibley School of Mechanical and Aerospace Engineering, Cornell University, Ithaca, NY USA

**Keywords:** Meniscus, Tissue engineering, Regeneration, Repair, Collagen

## Abstract

**Background:**

Bone marrow mesenchymal stem cells (MSCs) have shown positive therapeutic effects for meniscus regeneration and repair. Preliminary in vitro work has indicated positive results for MSC applications for meniscus tissue engineering; however, more information is needed on how to direct MSC behavior. The objective of this study was to examine the effect of MSC co-culture with primary meniscal fibrochondrocytes (FCCs) in a three-dimensional collagen scaffold in fibrochondrogenic media. Co-culture of MSCs and FCCs was hypothesized to facilitate the transition of MSCs to a FCC cell phenotype as measured by matrix secretion and morphology.

**Methods:**

MSCs and FCCs were isolated from bovine bone marrow and meniscus, respectively. Cells were seeded in a 20 mg/mL high-density type I collagen gel at MSC:FCC ratios of 0:100, 25:75, 50:50, 75:25, and 100:0. Constructs were cultured for up to 2 weeks and then analyzed for cell morphology, glycosaminoglycan content, collagen content, and production of collagen type I, II, and X.

**Results:**

Cells were homogeneously mixed throughout the scaffold and cells had limited direct cell–cell contact. After 2 weeks in culture, MSCs transitioned from a spindle-like morphology toward a rounded phenotype, while FCCs remained rounded throughout culture. Although MSC shape changed with culture, the overall size was significantly larger than FCCs throughout culture. While 75:25 and 100:0 (MSC mono-culture) culture groups produced significantly more glycosaminoglycan (GAG)/DNA than FCCs in mono-culture, GAG retention was highest in 50:50 co-cultures. Similarly, the aggregate modulus was highest in 100:0 and 50:50 co-cultures. All samples contained both collagen types I and II after 2 weeks, and collagen type X expression was evident only in MSC mono-culture gels.

**Conclusions:**

MSCs shift to a FCC morphology in both mono- and co-culture. Co-culture reduced hypertrophy by MSCs, indicated by collagen type X. This study shows that MSC phenotype can be influenced by indirect homogeneous cell culture in a three-dimensional gel, demonstrating the applicability of MSCs in meniscus tissue engineering applications.

**Electronic supplementary material:**

The online version of this article (doi:10.1186/s13287-016-0301-8) contains supplementary material, which is available to authorized users.

## Background

Meniscus damage is one of the most common knee injuries with a reported incidence of 61/100,000 and over 1 million procedures performed annually in the United States [1]. Like most cartilaginous structures, the meniscus has a limited healing capacity because the tissue is primarily avascular. Depending on the severity of the tear, the meniscus is either partially resected or replaced using a meniscus allograft. Meniscal allograft procedures are limited in availability, shape and immunocompatibility [2–4]. Recent studies have demonstrated the applicability of intra-articular stem cell injection for repair of small meniscal tears [5–8]. For more extensive injuries, tissue engineering of the meniscus may offer a promising alternative to meniscus allograft replacement.

Progress toward a tissue-engineered meniscus has shown great promise, but has yet to reach clinical application [9–13]. Tissue-engineered menisci often lack native biochemical and mechanical properties necessary for successful function in vivo. The addition of cells to engineered menisci provides an essential mediator for development and modification of the construct, often resulting in a better match to native properties. Previously, we have shown that fibrochondrocytes (FCCs) seeded in a collagen tissue-engineered meniscus under static mechanical boundary conditions are able to mimic anisotropic fiber formation seen in native menisci as well as improve mechanical properties [14]. However, obtaining the sufficient number of cells for an engineered meniscus is challenging. FCCs derived from surgical debris have been shown as a viable cell source for tissue engineering [15], but they remain a challenge since cell number is limited, as FCCs proliferate slowly and often lose their phenotype in two-dimensional (2D) culture [16]. As tissue engineered constructs approach clinical application there is an increasing need for a cell source that is easy to obtain and expand in culture.

Mesenchymal stem cells (MSCs) have shown great potential as a treatment option for meniscus repair and regeneration. Intra-articular injection of bone marrow-derived MSCs in human and animal studies have demonstrated that MSCs mobilize to the site of injury and contribute to tissue regeneration [5–8]. MSCs from the bone marrow are easily obtained and expanded in culture and are well established as multipotent stem cells that can differentiate down chondrogenic lineage [17, 18]. However, differentiation of MSCs down the fibrochondrogenic lineage is not well understood [19]. MSC co-culture with either meniscus or articular cartilage cells has been shown to direct differentiation and increase matrix secretion [20–24]. Pellet culture of MSCs co-cultured with meniscus FCCs increased expression of fibrochondrogenic genes, reduced hypertrophy, and increased matrix production [20, 21]. However, studies using FCC co-culture with stem cells have been limited to 2D culture and three-dimensional (3D) cell pellets. Little is known about how cell proximity, exogenous signaling, and cell–matrix interactions will affect cellular phenotype in 3D scaffold culture.

The goal of this study was to evaluate the effects of 3D co-culture of MSCs and FCCs on cell phenotype indicated by cell shape, matrix secretion, and mechanical properties of constructs. We hypothesize that co-culture of MSCs with FCCs in a 3D collagen scaffold will facilitate increased matrix secretion and mechanical properties.

## Methods

### Cell isolation

Methods for cell isolation were based on those previously described, in which all cells were isolated from 1- to 3-day-old bovids postmortem [25, 26]. Briefly, MSCs were extracted by washing the trabecular region of the femoral head with heparin supplemented media [26]. The extract solution was centrifuged at 300 × *g* and the pellet was suspended and plated on tissue culture plastic. Plates were washed after 48 hours to remove the unattached cell population. Trilineage differentiation assays were performed to confirm multipotency of MSCs for ostegenicity, adipogenicity, and chondrogenicity (Additional file 1) [18, 27]. MSCs were plated at 2000 cells/cm^2^ and expanded in 2D culture until passage 4 with a growth medium containing low glucose Dulbecco’s modified Eagle’s medium (DMEM) supplemented with 10 % fetal bovine serum (FBS), 100 IU/mL penicillin, 100 μg/mL streptomycin, 0.25 μg/mL amphotericin B, 2 mM L-glutamine, and 1 ng/mL basic fibroblast growth factor. FCCs were digested from menisci in 0.3 % collagenase (Worthington Biochemical Corporation, Lakewood, NJ, USA) in DMEM with 100 μg/mL penicillin and 100 μg/mL streptomycin, followed by filtering through a 100-μm cell strainer [25, 28]. Following cell isolation, FCCs were prepared for direct seeding into collagen gels with passaged MSCs. Prior to mixing cells into 3D constructs, MSCs were labeled using CellTrace Green CFSE (Invitrogen, Grand Island, NY, USA; C34554) and FCCs were labeled with CellTrace FarRed DDAO-SE (Invitrogen; C34553). Cell media cocktails were mixed at MSC:FCC ratios of 0:100, 25:75, 50:50, 75:25, and 100:0. Since no live animals were used in this study, no IACUC approval was required.

### Construct generation

Collagen type I was extracted from Sprague–Dawley rat tails (Pel-Freez Biologicals, Rogers, AZ, USA) and reconstituted in 0.1 % acetic acid at 30 mg/mL concentration as previously described [25, 29, 30]. Briefly, the stock collagen solution was mixed with working solutions of 1N NaOH, 10× phosphate-buffered saline (PBS), and 1× PBS to return the collagen to a neutral 7.0 pH and 300 mOsm and begin the gelation process [30]. Cell-media cocktails were homogeneously mixed at a final concentration of 25 × 10^6^ cells/mL to form a collagen solution at 20 mg/mL [25]. Collagen solution was gelled between two glass plates to create a sheet gel 2 mm thick, and molds were allowed to gel for 30 minutes at 37 °C. From each 2-mm thick gel, 30 8-mm diameter samples were obtained using biopsy punches. Ten samples were used per time point at 1, 8, and 15 days (two to confocal/histology, four to mechanical, and four to biochemical analysis). Samples were cultured in media containing DMEM, 10 % FBS, 100 μg/mL penicillin, 100 μg/mL streptomycin, 0.1 mM non-essential amino acids, 50 μg/mL ascorbate, and 0.4 mM L-proline [25]. Culture media was collected and replenished every 3–4 days. Images of each sample were obtained at each media change. Images were imported into ImageJ to calculate the area of each construct. Cells and constructs were cultured at 37 °C and 5 % CO_2_.

### Cell shape analysis

At the desired time points, two samples from each experimental group were fixed in 10 % buffered formalin for 48 hours and stored in 70 % ethanol. Fluorescence imaging was performed on a Zeiss 710 confocal microscope with a Zeiss Axio Observer Z1 inverted stand using a 40×/1.2 C-Apochromat water immersion objective. Images of MSCs labeled with CellTrace Green CFSE and FCCs labeled with CellTrace FarRed DDAO-SE were obtained separately for analysis. Four images and two z-stacks per sample were taken, with at least ten cells per image. Z-stacks were converted into a 2D projected image. Aspect ratio (AR) and cell area were calculated using “area” and “centroid fit” (AR = major axis/minor axis) in ImageJ software (National Institute of Health) [31].

### Biochemical content

Samples were collected and weighed to obtain a wet weight (WW), then frozen, lyophilized, and weighed again to obtain dry weight. As previously described, DNA, glycosaminoglycan (GAG), and collagen content were measured via the Hoechst DNA assay [32], a modified 1,9-dimethylmethylene blue (DMMB) assay at pH 1.5 [33], and a hydroxyproline (hypro) assay, respectively [34]. Biochemical contents were normalized to DNA to account for construct contraction and cell proliferation. Biochemical tests were analyzed on both construct samples and media samples collected throughout culture. Total content was calculated as a sum of biochemical content in media added to total biochemical content in the construct. Retention was calculated as a percentage of content in construct relative to total content.

### Histology

Following fluorescent imaging, samples were dehydrated, embedded into paraffin blocks, sectioned, and stained. Picrosirious red staining was imaged using brightfield microscopy and collagen fiber organization was visualized under polarized light [25]. Immunohistochemistry was conducted as previously described to further investigate collagen content using antibodies for collagen type I (Abcam, Cambridge, MA, USA; 34710), collagen type II (Chondrex, Redmond, WA, USA; 7005), and collagen type X (Abcam; 58632) [35]. Primary and secondary antibody controls were run in parallel with samples for immunohistochemistry stains (Additional file 2). Control samples and experimental samples were stained in the same batch process and exposed to the same duration and concentration of reagents. Images were obtained with a SPOT RT camera (Diagnostic Instruments, Steriling Heights, MI, USA) attached to a Nikon Eclipse TE2000-S microscope (Nikon Instruments, Melville, NY, USA).

### Mechanical properties

Four samples per experimental group were cut into 4-mm diameter plugs and tested for compressive properties [36–38]. Samples (2-mm thick) were tested in confined compression via a stress relaxation test performed by imposing 10 × 100 μm steps (relaxation = 12 minutes, strain = 5–45 %, steps = 5 %, n = 4). The resulting load was then fit to a poroelastic model using a custom MATLAB program to determine aggregate modulus (HA) and hydraulic permeability (k). Mechanical testing was performed on an Enduratec ElectroForce 3200 System (Bose, Eden Prairie, MN, USA) using a 1-kg load cell.

### Statistics

Biochemical data were analyzed by two-way analysis of variance using Tukey’s t-test for post-hoc analysis (SigmaPlot, San Jose, CA, USA). An equal probability averaging method was used for GAG retention calculations to pair media samples with construct samples [39]. All data are expressed as mean ± standard deviation and significance was determined with *p* < 0.05.

## Results

### Characterization of cell morphology

**Fig. 1:**
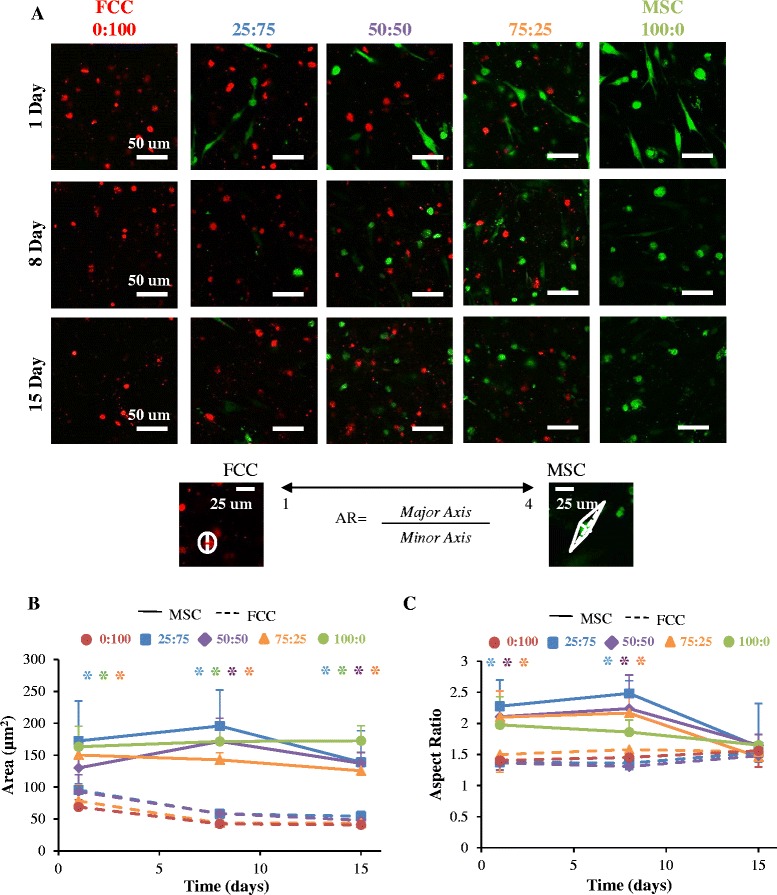
Cell shape and size images with analysis with all ratios presented as MSC:FCC. **a** Fluorescent images of biopsy constructs (*red* = FCC, *green* = MSC); scale bar = 50 μm, n = 2. **b** Cell area calculations and **c** aspect ratio calculations (*dotted line* represents FCC population in co-culture and *solid line* represents MSC cell population in co-culture). *p < 0.05, versus 100 % FCC; n = 4. *AR* Aspect ratio, *FCC* fibrochondrocyte, *MSC* mesenchymal stem cell

Cells embedded in collagen gels visualized using fluorescent probes showed that cells were homogeneously distributed within the construct, with limited direct cell–cell contact. Within each construct, cells were homogenously mixed between the two cell types, with FCCs and MSCs distributed throughout the construct (Fig. 1a).

MSCs had an increased projected area relative to FCCs, with MSCs (mono-culture area = 169 ± 119 μm^2^) approximately two-times the size of FCCs (mono-culture area = 75 ± 40 μm^2^) at day 1. FCCs became smaller in area after 8 days (mono-culture area = 42 ± 13 μm^2^) but maintained size between 8 and 15 days (mono-culture area = 45 ± 18 μm^2^). After 15 days in culture, MSCs had reduced in size (mono-culture area = 162 ± 90 μm^2^); however, they were still significantly larger than the FCC cell population (Fig. 1b).

MSCs and FCCs exhibited distinct cell morphologies at day 1 that became more homogeneous after 15 days of culture (Fig. 1). FCCs appeared more rounded (mono-culture AR = 1.4 ± 0.3) and MSCs appeared more elongated (mono-culture AR = 2.0 ± 1.2) at day 1 (Fig. 1c). MSC cell shape at day 1 was a mixture of circular and elongated cell morphologies. After 15 days in culture, both FCCs (mono-culture AR = 1.5 ± 0.4) and MSCs (mono-culture AR = 1.7 ± 0.6) displayed a circular morphology (Fig. 1a). FCC AR remained consistent between experimental groups in co-culture and throughout the duration of co-culture with no statistical differences. MSCs showed variable morphologies at 1 and 8 days, and appeared to converge on the circular phenotype after 15 days (Fig. 1a and c).

### Matrix synthesis

**Fig. 2:**
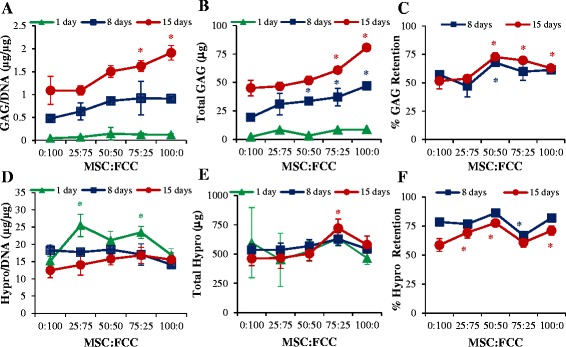
Biochemical analysis with all ratios presented as MSC:FCC. **a** Glycosaminoglycan (*GAG*) content in construct normalized to DNA content. **b** Total GAG produced by sample calculated as a sum of GAG in the media and GAG in the construct. **c** GAG retained within construct calculated as a percentage of content in samples to total content in samples and media over time. **d** Hydroxyproline (*hypro*) content in construct normalized to DNA content. **e** Total hypro produced by sample calculated as a sum of GAG in the media and GAG in the construct. **f** Hypro retained within construct calculated as a percentage of content in samples to total content in samples and media over time. *p < 0.05, versus 100 % (0:100) FCC within time point; n = 4. *FCC* Fibrochondrocyte, *MSC* mesenchymal stem cell

Phenotypic changes were observed in MSC-laden gels as measured through changes in matrix content over time. Gels contained essentially no GAG at the beginning of culture. After fifteen days, GAG normalized to DNA content increased to 1.08 ± 0.3 μg/μg in FCC mono-culture and to 1.91 ± 0.16 μg/μg in MSC mono-culture. GAG/DNA content increased linearly with MSC content at fifteen days (R^2^ = 0.94) and all groups had a significant increase in GAG/DNA content with time (Fig. 2a; *p* < 0.001). GAG content in media was recorded in order to observe if cells were producing GAGs that were being lost into the cell media. The increasing GAG/DNA content increasing with MSC content remained consistent when GAG in media is combined with GAG in the construct (Fig. 2b). Interestingly, 50:50 co-culture retained the greatest amount of GAG within the construct (73 ± 3 %), significantly higher than both FCC and MSC mono-culture. MSC gels at 50:50, 75:25, and 100:0 all retained significantly higher amounts of GAG (73 ± 3 %, 70 ± 2 %, and 63 ± 2 %, respectively) compared to FCC mono-culture (52 ± 7 %; Fig. 2c). Hydroxyproline was measured as an indication of collagen content. All gels contained collagen at 1 day since the gels were comprised of collagen type I; however, over time the cells break down their collagen matrix. FCCs displayed a more catabolic response than the MSCs as the MSC mono-culture group had no significant changes in hypro/DNA (Fig. 2d). Hydroxyproline content measured in the media and construct together indicated that the total collagen in the system is not changing with time (Fig. 2e). Similar to GAG retention, hydroxyproline retention in the constructs was greatest in the 50:50 co-culture group (77 ± 3 %; Fig. 2f). Collagen constructs contracted over time, but maintained a cylindrical shape (Additional file 3A). MSC-containing constructs contracted between 40–60 % of their original size by day 15, while FCC mono-culture gels contracted only to 82 % of the original size (Additional file 3B). There was no significant difference between gels; therefore, contraction does not play a role in biochemical differences between these groups. Cells in constructs proliferated between days 1, 8, and 15, indicating a healthy cell population. DNA content between groups at day 1 showed no significant differences; however, co-culture and MSC mono-culture groups had greater proliferation than FCC mono-culture over time (Additional file 3C). GAG and hydroxyproline content were normalized to DNA to account for cellular proliferation with time.

**Fig. 3:**
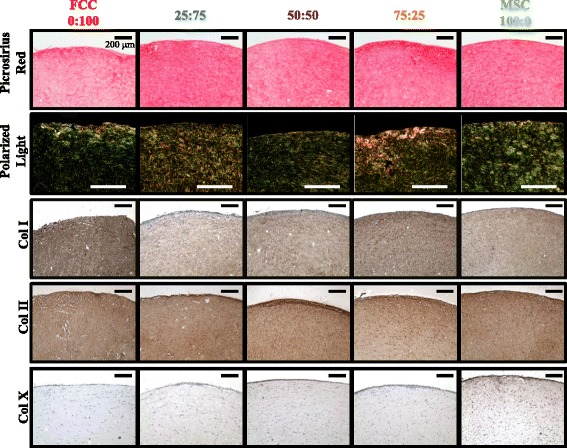
Histological staining of samples after 15 days of culture with all ratios presented as MSC:FCC. Picrosirius red staining imaged with brightfield microscopy (*row 1*) and polarized light (*row 2*). Immunohistochemical staining of collagen type I (*row 3*), collagen type II (*row 4*), and collagen type X (*row 5*). Counterstained with hematoxylin; scale bars = 200 μm. *Col*, Collagen, *FCC* fibrochondrocyte, *MSC* mesenchymal stem cell

Histological staining and immunochemistry revealed matrix presence and localization. All experimental groups showed the presence of collagen with small disorganized fibers forming in the body of the constructs. Small clumps of fibers formed throughout the construct with some increased alignment occurring near the edges (Fig. 3, rows 1 and 2). Immunohistochemistry was used to probe for specific types of collagen in the constructs. All groups stained positive for collagen type I and II after 15 days (Fig. 3, rows 3 and 4). No staining for collagen type II was observed in day 1 samples; thus, positive staining for collagen type II at 15 days was produced during culture (Additional file 4). After 2 weeks of culture, MSC mono-culture gels showed positive staining for collagen type X compared to other culture groups (Fig. 3, row 5).

### Mechanical characterization

**Fig. 4:**
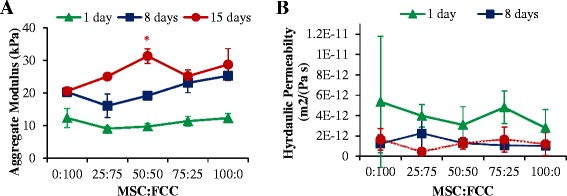
Mechanical analysis with all ratios presented as MSC:FCC. **a** Aggregate modulus and (**b**) permeability of constructs. *p < 0.05, versus 100 % FCC; n = 3–4. *FCC* Fibrochondrocyte, *MSC* mesenchymal stem cell

Mechanical properties of samples improved with MSC cellular content and time in culture (HA values, *p* < 0.05). Aggregate modulus of 50:50 co-culture (31 ± 2 kPa) was significantly higher that FCC mono-culture after 15 days. FCCs had the lowest aggregate modulus after 15 days in culture (21 ± 1 kPa) (Fig. 4a). Permeability reflected a similar trend to aggregate modulus with permeability decreasing from day 1 in culture; however, there was no statistical difference between sample groups at 15 days (Fig. 4b).

## Discussion

The objective of this study was to examine MSC phenotype when co-cultured with FCCs with the overarching goal to examine regenerative potential of MSCs for meniscus repair. We hypothesized that co-culture of MSCs with FCCs in a 3D collagen scaffold would facilitate increased matrix accumulation and mechanical properties. In this study, MSCs mono-cultured and co-cultured with FCCs displayed a phenotypic change related to cell morphology and matrix production. MSCs transition to a chondrogenic morphology and outperform FCCs in GAG production after 15 days of culture. Despite the advantageous matrix synthesis of MSCs, they had a hypertrophic tendency that was mitigated by co-culture. These data show that MSCs in co-culture with meniscal FCCs present specific advantages for meniscus tissue engineering, specifically increasing GAG retention in the construct, decreasing MSC hypertrophy, and improving mechanical properties.

Cell aspect ratio is well established as a measure of cell morphology and cell phenotype; however, this is the first study to examine changes in MSC morphology in co-culture with FCCs. In this study, MSCs underwent a distinct change in cell morphology between 1, 8, and 15 days. FCCs exhibited a consistent circular morphology, while MSCs started with an elongated morphology that transitioned to a circular morphology over time. Cell shape is directly linked to cell phenotype and has been shown to be dictated by the surrounding mechanical and chemical environment [40–42]. Reduction in cell spreading and/or transition to circular phenotype is associated with mesenchymal chondrogenesis. Previous work has shown that prevention of cell spreading, through disruption of the cytoskeleton using cytochalasin, increased chondrogenesis [43]. Pellet cultures are hypothesized to aid in MSC chondrogenesis by providing a 3D environment that forces cells into a compacted shape, reducing cell spreading [18]. In this study, cultured MSCs, even in a material that supports adhesion and spreading, moved to a rounded phenotype and produced proteoglycans.

Consistent with other studies, we found that MSCs increased collagen type X expression in mono-culture and that this response was mitigated in co-culture. These MSCs originate from bone marrow and are known to have similar functional behavior to growth plate chondrocytes which express the hypertrophic phenotype [44]. Co-culture has been shown in multiple studies to mitigate hypertrophic effects both with chondrocytes [22, 24, 26] and, to a lesser extent, with fibrochondrocytes [20, 21]. Previous studies focused on evaluating collagen type X gene expression as a marker of hypertrophy, whereas we measured collagen type X presence in constructs using immunohistochemistry. Hypertrophy is also characterized by an enlargement of cell area and volume. In this study we measure cell area and observed that MSCs were nearly twice the size of FCCs, but there was no significant enlargement of cells with time in culture. Although not significant, the MSC fraction of cells in co-culture groups showed a reduction in area after 15 days, while MSCs in mono-culture showed a slight increase in area, indicating a more hypertrophic cell population in MSC mono-culture [45]. This study supports the body of work that suggests that co-culture of MSCs with chondrocytes or FCCs is a mechanism for functional inhibition of MSC hypertrophy.

Cell–material interactions are known to influence changes in cellular behavior and phenotype. Collagen was the primary scaffold material in our gels, which is known to influence cell phenotype through both chemical and mechanical pathways. Similar to previous pellet culture studies of MSC and FCC mono- and co-culture [20, 21], GAG and collagen type II were increased in all culture groups in meniscal media. The FCC mono-culture gel in this study appeared to have an increased collagen type I expression compared to gels with MSCs. The meniscus is composed primarily of collagen type I, with collagen type II being the second most prominent collagen, especially in the cartilaginous inner region [46, 47]. The MSC containing groups did produce more GAG in construct which is consistent with a more chondrogenic behavior and less collagen type I production seen in the inner zone of the meniscus [48]. In this study, the MSC mono-culture group produced the most GAG, in contrast to other studies in which MSC mono-cultures usually contain the lowest GAG concentration [20, 21, 26, 49]. This discrepancy is likely due to cell–material interactions which provide a physical diffusion barrier that is lacking in pellet culture. Previous studies using alginate showed that MSCs produced more GAG in mono-culture than FCCs, but were unable to retain GAG within the construct [50]. Another study showed that MSCs cultured on a collagen scaffold had increased expression of collagen type II, sox9, and aggrecan expression compared to alginate [51]. Furthermore, a study investigating the effect of articular chondrocytes co-cultured with MSCs in pellet culture versus within a collagen type I scaffold demonstrated that GAG/WW content was lowest in FCC mono-culture group in collagen, whereas in pellet culture the FCC group contained the greatest amount on GAG/DNA [52]. This study is the first to examine MSC and FCC co-culture in a collagen gel and supports that the matrix used to culture MSCs is an important contributor to guiding MSC phenotype.

Previous studies attribute phenotypic changes to close cellular proximity in pellet cultures; however, our study demonstrated that direct cell–cell contact is not necessary for phenotypic changes in MSC behavior. Studies in pellet culture have noted increased matrix expression in co-cultures compared to mono-culture controls, which could be attributed to direct cell–cell contact resulting in an interaction effect [20, 21]. Furthermore, pellet culture may promote chondrogenesis because cells are forced into a compact circular phenotype rather than allowing them to spread on a surface [18]. Previous studies have shown that stem cell differentiation can be controlled by soluble signaling factors [18, 48, 53]. Specifically, conditioned media from chondrocytes directed chondrogenic differentiation of MSCs and enhanced matrix production [54, 55]. A modeling study concluded that a single cell can effectively communicate within a domain of 250 μm [56]. Another study demonstrated that soluble effects require close proximity because increased matrix and mechanical properties were only seen in co-cultured MSC and chondrocyte hydrogels as opposed to two distinct hydrogels cultured in the same well [24]. In this study, there was increased matrix expression of GAG and collagen type I in constructs. Of particular interest was that the 50:50 culture group had the highest GAG retention. Previously, we have shown that MSCs in mono-culture are deficient in link protein compared to chondrocyte cells which resulted in a loss of GAG into media in MSC constructs [50]. Our co-cultured groups likely had the advantage of FCC production of link protein to retain the increased production of GAG from MSCs. This study supports that MSC phenotypic changes do not require direct cell–cell contact, suggesting that soluble signaling factors play a key role in directing phenotypic changes.

This study was the first to show mechanical evaluation of constructs using FCC and MSC co-culture. Conducting these studies in a 3D scaffold enabled the measurement of mechanical properties to quantify effects that cellular remodeling and matrix production had on mechanical properties. MSCs and FCCs cultured in collagen gels stiffened with time in all groups. The 50:50 co-culture showed the greatest increase in compressive properties, with 100:0 MSC mono-cultures showing a similar increase. The 100:0 MSC mono-culture had the greatest GAG/DNA production; however, 50:50 co-culture had the greatest GAG retention. Previously we have shown that compressive mechanical properties are not exclusively correlated with GAG content and that collagen content is particularly important for the compressive properties of meniscal constructs [25, 28]. Furthermore, the mechanical properties of a substrate are a key factor contributing to MSC fate [40, 57]. Increasing construct stiffness in a 3D gel likely contributed to phenotypic changes in gels toward chondrogenic morphology and matrix expression.

This study has some limitations. The stem cells used in this study were not tested and sorted for cell surface antigens and are therefore a heterogeneous population. However, MSCs used in this study were characterized and validated by two well-established defining criteria: plastic adherence and trilineage differentiation. The protocol used in this study has been well established in previous literature to yield viable stem cells [26]. Bovine MSCs and FCCs were used for the purposes of these experiments. The use of bovines as a cell source could affect clinical translatability of experiments. The 50:50 co-culture showed the best mechanical properties and GAG retention; however, obtaining 50 % FCCs may not be clinically feasible.

## Conclusion

This study shows that MSC phenotype can be influenced by co-culture in a 3D dimensional construct. MSCs demonstrated a transition to chondrogenic phenotype supported by changes in cell shape, matrix production, and mechanical properties. Maximal mechanical performance and GAG retention was observed in the 50:50 co-culture group. Additionally, co-culture groups showed reduced hypertrophy to MSCs in mono-culture. While the specific cause of MSC differentiation remains unknown, this study validates that MSCs in 3D scaffold co-culture transition to FCC phenotype, demonstrating their applicability for 3D tissue-engineered menisci as well as other tissue engineering applications.

## Additional files

Additional file 1: Trilineage differentiation of bone marrow derived MSCs. Control samples cultured in growth medium and stained with Alizarin Red S, Oil Red O or Safranin O. Samples cultured in osteogenic media stained with Alizarin Red, samples cultured in adpogenic media stained with Oil Red O and samples cultured in chondrogenic media stained with Safranin O. (PPTX 120 kb) Additional file 2: Immunohistochemicial staining controls for collagen type I, II, X. Primary antibody controls run for all groups (−). Extensor tendon section from bovine knee as positive control for collagen type I. Articular cartilage transverse section from bovine distal femur as positive control for collagen type II. Growth plate from bovine distal femur as positive control for collagen type X. Counterstained with hematoxylin; scale bar on 0 week collagen gel = 200 μm on 100× objective; all other scale bars = 500 μm on 40× objective). (PPTX 3053 kb) Additional file 3: (A) Macroscopic images of constructs taken at each time point and condition. (B) Projected area calculations of samples over time. (C) DNA content normalized to wet weight of samples. **p* < 0.05, versus 100 % FCC; n = 4. (PPTX 513 kb) Additional file 4: Immunohistochemicial staining for collagen type II. Counterstained with hematoxylin; scale bar = 200 μm. (PPTX 1035 kb)

## Abbreviations

2D: Two-dimensional
3D: Three-dimensional
AR: Aspect ratio
DMEM: Dulbecco’s modified Eagle’s medium
FBS: Fetal bovine serum
FCC: Fibrochondrocyte
GAG: Glycosaminoglycan
HA: Aggregate modulus
Hypro: Hydroxyproline
k: Hydraulic permeability
MSC: Mesenchymal stem cell
PBS: Phosphate-buffered saline
WW: Wet weight
