# Supplementary figures and images for: Characterization of mesenchymal stem cells and fibrochondrocytes in three-dimensional co-culture: analysis of cell shape, matrix production, and mechanical performance
Source: Stem Cell Res Ther. 2016 Mar 12;7:39. doi: 10.1186/s13287-016-0301-8 (PMC4789279; doi:10.1186/s13287-016-0301-8)

## Slide 1
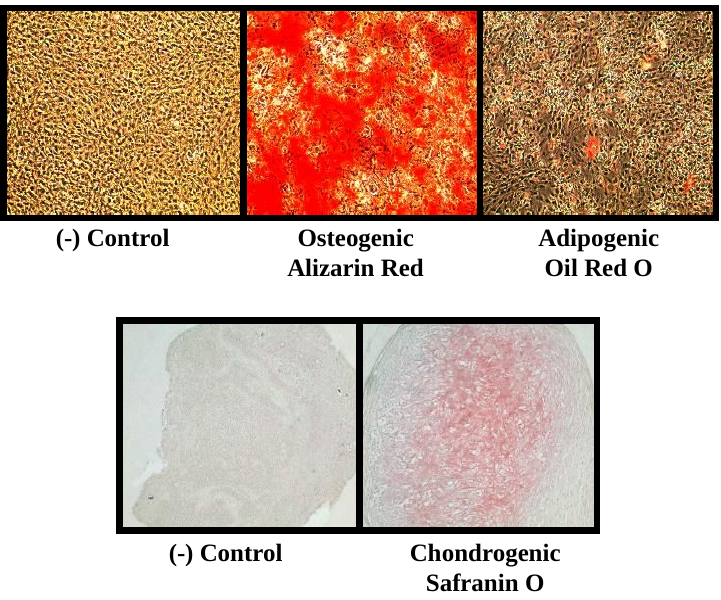

(-) Control
Osteogenic
Alizarin Red
Adipogenic
Oil Red O
(-) Control
Chondrogenic
Safranin O

Supplement: Additional file 1: — Trilineage differentiation of bone marrow derived MSCs. Control samples cultured in growth medium and stained with Alizarin Red S, Oil Red O or Safranin O. Samples cultured in osteogenic media stained with Alizarin Red, samples cultured in adpogenic media stained with Oil Red O and samples cultured in chondrogenic media stained with Safranin O. (PPTX 120 kb) [file 13287_2016_301_MOESM1_ESM.pptx]

## Slide 1
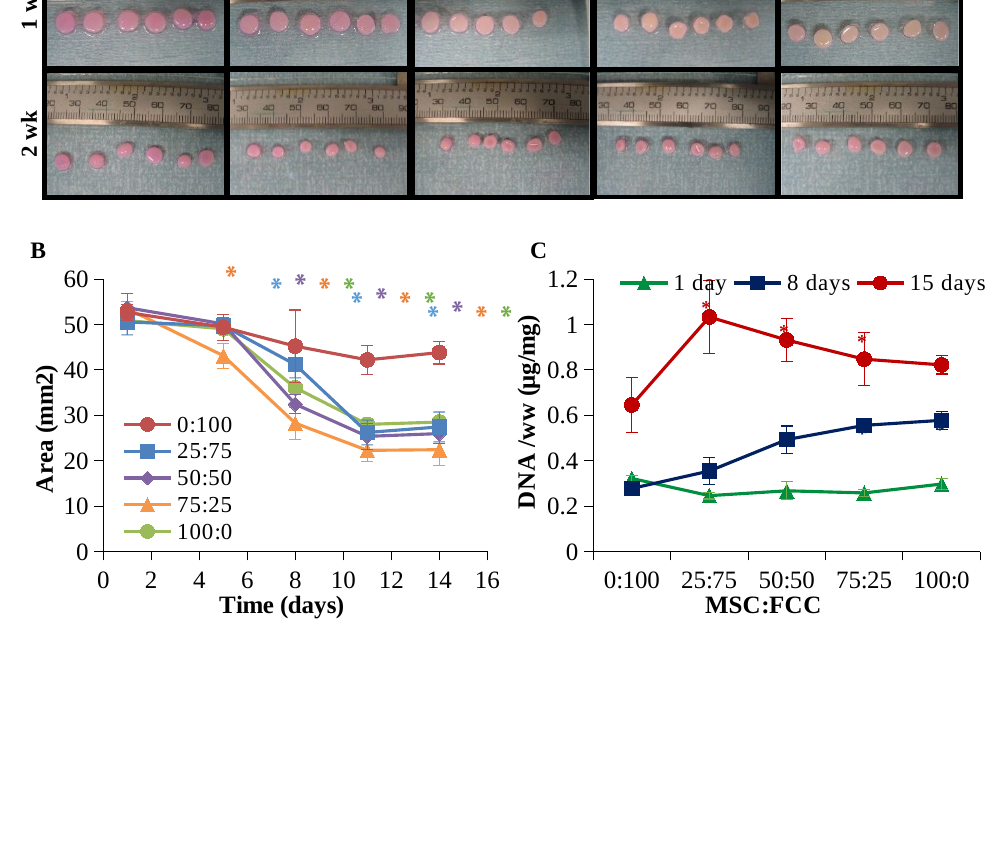

A
FCC
0:100
MSC
100:0
50:50
75:25
25:75
0 wk
1 wk
2 wk
*
*
*
*
B
C
*
*
*
*
*
*
*
*
*
### Chart
| Category | 0:100 | 25:75 | 50:50 | 75:25 | 100:0 |
|---|---|---|---|---|---|
### Chart
| Category | | | |
|---|---|---|---|
| 0:100 | 0.3220788737769202 | 0.27823649364197084 | 0.6455350415069676 |
| 25:75 | 0.24662009656500183 | 0.3549632513419123 | 1.0328954782928188 |
| 50:50 | 0.2677300115826975 | 0.49318909009648226 | 0.9317570102973812 |
| 75:25 | 0.2584800289868329 | 0.5557934449136326 | 0.8472149673612407 |
| 100:0 | 0.2976746095913723 | 0.5780526902850968 | 0.8220483608842265 |*
*
*
*
*
*
*
*

Supplement: Additional file 3: — (A) Macroscopic images of constructs taken at each time point and condition. (B) Projected area calculations of samples over time. (C) DNA content normalized to wet weight of samples. *p < 0.05, versus 100 % FCC; n = 4. (PPTX 513 kb) [file 13287_2016_301_MOESM3_ESM.pptx]

## Slide 1
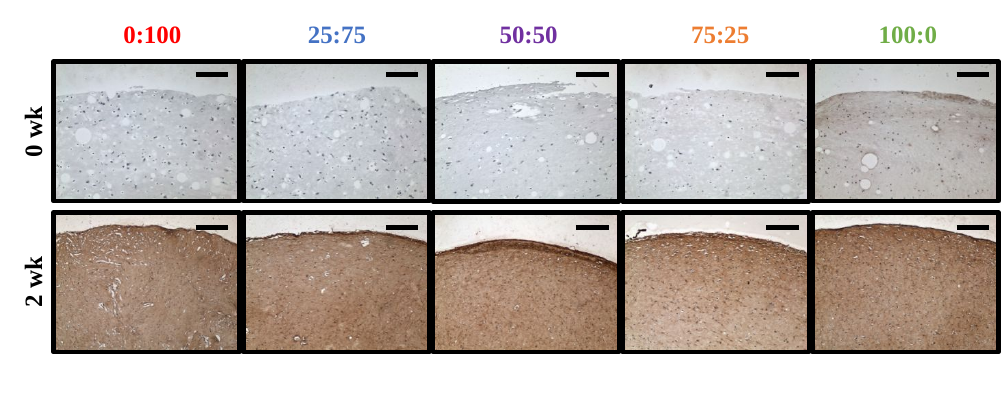

0:100
25:75
50:50
75:25
100:0
0 wk
2 wk

Supplement: Additional file 4: — Immunohistochemicial staining for collagen type II. Counterstained with hematoxylin; scale bar = 200 μm. (PPTX 1035 kb) [file 13287_2016_301_MOESM4_ESM.pptx]
