# Supplementary material for: Characterization of mesenchymal stem cells and fibrochondrocytes in three-dimensional co-culture: analysis of cell shape, matrix production, and mechanical performance
Source: Stem Cell Res Ther. 2016 Mar 12;7:39. doi: 10.1186/s13287-016-0301-8 (PMC4789279; doi:10.1186/s13287-016-0301-8)
Supplement: Additional file 2: — Immunohistochemicial staining controls for collagen type I, II, X. Primary antibody controls run for all groups (−). Extensor tendon section from bovine knee as positive control for collagen type I. Articular cartilage transverse section from bovine distal femur as positive control for collagen type II. Growth plate from bovine distal femur as positive control for collagen type X. Counterstained with hematoxylin; scale bar on 0 week collagen gel = 200 μm on 100× objective; all other scale bars = 500 μm on 40× objective). (PPTX 3053 kb) [file 13287_2016_301_MOESM2_ESM.pptx]

## Slide 1
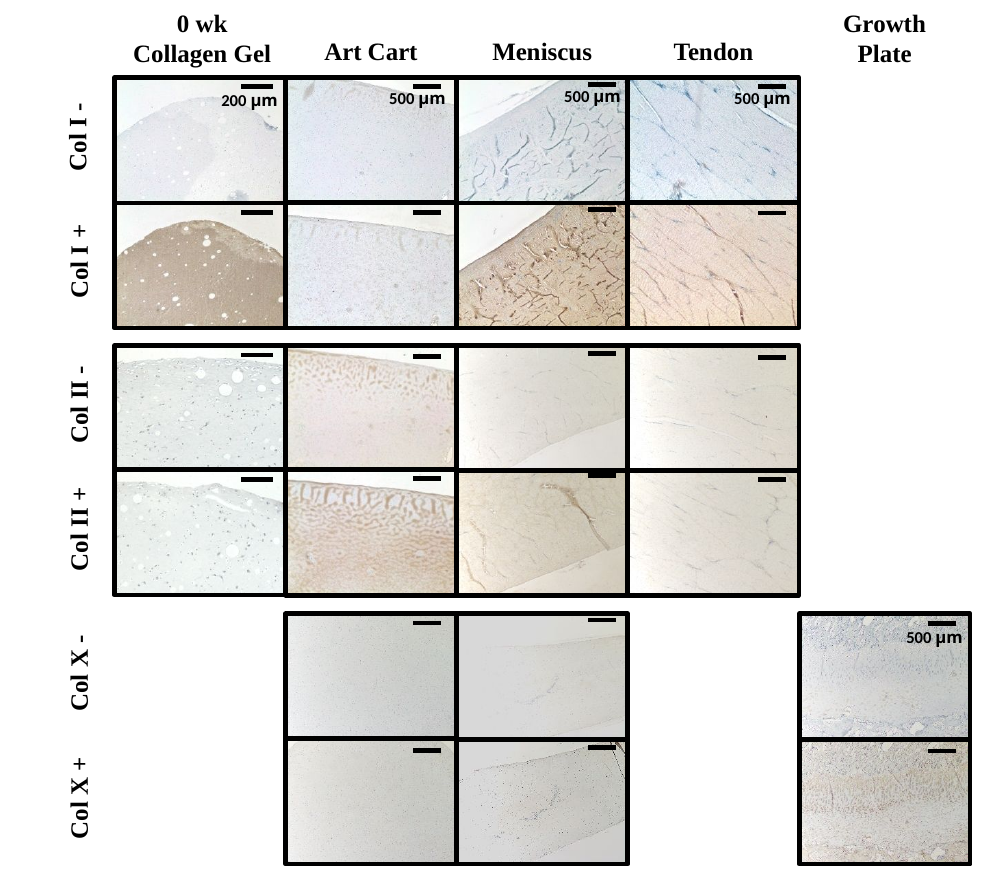

0 wk Collagen Gel
Growth Plate
Art Cart
Meniscus
Tendon
500 μm
500 μm
500 μm
200 μm
Col I -
Col I +
Col II -
Col II +
500 μm
Col X -
Col X +
